# Psychosocial factors associated with poor outcomes after amputation for complex regional pain syndrome type-I

**DOI:** 10.1371/journal.pone.0213589

**Published:** 2019-03-13

**Authors:** Ernst Schrier, Jan H. B. Geertzen, Jelmer Scheper, Pieter U. Dijkstra

**Affiliations:** 1 University of Groningen, University Medical Center Groningen, Department of Rehabilitation Medicine, Groningen, The Netherlands; 2 University of Groningen, University Medical Centre Groningen, Department of Oral and Maxillofacial Surgery, Groningen, The Netherlands; Holland Bloorview Kids Rehabilitation Hospital, CANADA

## Abstract

**Background:**

Amputation for longstanding therapy resistant complex regional pain syndrome type-I (CRPS-I) is controversial. Reported results are inconsistent. It is assumed that psychological factors play a role in CRPS-I.

**Objective:**

To explore which psychological factors prior to amputation are associated with poor outcomes after amputation in the case of longstanding therapy resistant CRPS-I.

**Methods:**

Between May 2008 and August 2015, 31 patients with longstanding therapy resistant CRPS-I were amputated. Before the amputation 11 psychological factors were assessed. In 2016, participants had a structured interview by telephone and filled out questionnaires to assess their outcome. In case of a perceived recurrence of CRPS-I a physician visited the patient to examine the symptoms. Associations between psychological factors and poor outcomes were analysed.

**Results:**

Four of the 11 psychological factors were associated with poor outcomes. Regression analyses showed that change in the worst pain in the past week was associated with poor social support (B = 0.3, 95% confidence interval: 0.1;0.6) and intensity of pain before amputation (B = 2.0, 95% confidence interval 0.9;3.0). Patients who reported important improvements in mobility (n = 23) had significantly higher baseline resilience (median 79) compared to those (n = 8) who did not report it (median 69)(Mann-Whitney U, Z = -2.398, p = 0.015). Being involved in a lawsuit prior to amputation was associated with a recurrence in the residual limb (Bruehl criteria). A psychiatric history was associated with recurrence somewhere else (Bruehl criteria).

**Conclusion:**

Poor outcomes of amputation in longstanding therapy resistant CPRS-1 are associated with psychological factors. Outstanding life events are not associated with poor outcome although half of the participants had experienced outstanding life events.

## Introduction

Complex regional pain syndrome type-I (CRPS-I) is characterized by severe pain, sensory, vasomotor, sudomotor and trophic changes and can have a devastating effect on a person.[1] CRPS-I generally develops after an injury but sometimes it develops spontaneously. Many treatments have been described but only a few are evidence based.[2] Amputation in the case of longstanding therapy resistant CRPS-I is rare and controversial. It is rare because many patients with CRPS-I, recover within 6 to 13 months.[3] It is controversial because some patients benefit from the amputation, while others experience the same symptoms or even experience an increase of symptoms after the amputation.[4] These unpredictable outcomes make an amputation in longstanding therapy resistant CRPS-I debatable as treatment.[5] Hesitation to amputate is strengthened by the assumed role of psychological factors or psychiatric disorders in the aetiology, development and maintenance of CPRS-I.[6–10] However, data supporting this assumption are scant. In the University Medical Centre Groningen (UMCG) the decision to amputate or not is made by a team of specialists together with the patient.[11] For the psychologist, working in that team, a working hypothesis was that outcomes of an amputation would be negatively influenced by presence of some psychological factors: Poor Quality of Life (QOL) in the physical domain or psychological domain, low resilience, depression, anxiety, psychological distress, childhood adversity, life events, psychiatric (DSM-IV) history or psychiatric disorder, current lawsuit, and or poor social support.[12–14] In patients with an amputation for other causes, associations with poor QOL post amputation have been reported.[15–17] Poor QOL was associated with many factors including depression, social support, cognition, pain, independence in activities of daily living and comorbidity.[18, 19] Starting in May 2008 these factors were therefore routinely assessed during intake of patients who requested an amputation in the case of longstanding therapy resistant CRPS-I in our centre. Insight regarding which psychological factors are associated with poor outcomes could help the team to predict which patients suffering from longstanding therapy resistant CRPS-I should not be amputated. Current study is part of a larger outcome study of CRPS-I patients, amputated in the UMCG, starting in 2000. Of all the 48 patients participating in that study, 31 were assessed by a psychologist (ES) prior to amputation by means of a standardized interview and a set of questionnaires. The larger study focuses on several outcomes after amputation, assessed in 2015, but is cross-sectional in design. Focus of current study was to explore which psychological factors assessed prior to amputation are associated with poor outcomes after amputation.

As primary outcomes of this study change in pain and mobility after amputation were selected because most patients requested an amputation to improve on pain and or mobility. As a secondary outcome recurrence of CRPS-I was selected because after amputation recurrence in the residual limb or elsewhere is a major concern.[4, 5]

The aim of this longitudinal study was to analyse changes over time and to explore which psychological factors, present prior to amputation, were associated with poor outcomes after amputation in the case of longstanding therapy resistant CRPS-I.

## Methods

The research protocol was approved by the local Medical Research Ethics Committee (METc 2015/561) and all participants signed an informed consent before the start of the study.

Between May 2008 and august 2015, 33 adult patients with longstanding, therapy resistant CRPS-I underwent an amputation at the UMCG. CRPS-I was determined to be therapy resistant if all treatments described in the Dutch guidelines for CRPS-I had been tried.[20] Inclusion criteria for this follow-up study were: 18 years or older, participants should be able to comprehend questionnaires, and amputation was performed at least 1 year prior to follow-up. All 33 patients were asked to participate and all met inclusion criteria for this study. One patient did not respond and 1 patient had passed away. All participants met Bruehl criteria for CRPS-I at the time of amputation.[1]

More patients with longstanding therapy resistant CRPS-I requested an amputation at our Centre, but in about 50% of patients the requested amputation was refused. The main reasons to refuse were: criteria for CRPS-I were not met, patient expectations about the effects of an amputation were too optimistic (not realistic), the onset of CRPS-I was less than 1 year ago or all treatments described in the Dutch guidelines for CRPS-I had not yet been tried.[20]

Between May 2008 to August 2015, during the psychological assessment for the decision making process to amputate or not, a structured interview with the patient was performed. In that interview pain, childhood adversity, outstanding life events, a current lawsuit, a psychiatric disorder or history of a psychiatric disorder were assessed. Childhood adversity was operationalized as any experience(s), such as physical, mental or sexual abuse, occurring in childhood that cause(s) extreme stress. An outstanding life event was operationalized as any experience that caused stress far above the average. Additionally, a set of questionnaires was filled out.

In April 2016 an invitational letter to participate in this follow-up study was send to 33 patients. The follow-up study included a structured interview by telephone and filling out of questionnaires. Between May 30 2016 and August 11 2016 the structured interviews were held by a physician (JS), not involved in the decision making process of the amputation. Participants were also send a link to a secure website with the request to fill out a set of questionnaires. Attempts to acquire data were stopped January 1 2017.

In the interview, participants were asked to rate their worst and their least pain, in the past week, on a numeric rating scale (NRS): 0 = no pain and 10 = the worst imaginable pain. Participants were asked to rate their change in mobility after amputation, compared to the mobility prior to amputation, on a 5 point Likert scale (important improvement, small improvement, no change, small deterioration or important deterioration). If the participant reported a recurrence of CRPS-I, the physician (JS) visited the patient to evaluate recurrence according to Bruehl criteria.[20]

The following questionnaires were filled out prior to amputation and at follow-up.

The Quality of Life Questionnaire (WHOQOL-BREF) was used to assess quality of life in 4 different domains. It is a 26 item questionnaire that correlates well with the original 100 item questionnaire (r ranges from 0.88 to 0.96).[21] The WHOQOL-BREF has been field-tested widely.[22] In this study we used 3 domains of the questionnaire; physical health (7 items), psychological health (6 items) and social relationships (3 items). Raw data were transformed into domain scores range from 4 to 20 following the guidelines.[23] A higher score indicates a better QOL. The social relationships scale was used to determine social support. One question of this scale assesses satisfaction with support of friends and 1 assesses satisfaction with personal relationships. We operationalized poor social support as a score 1SD below the mean of all participants.

The Connor-Davidson Resilience Scale (CD-RISC), a 25 item questionnaire, was used to evaluate resilience. Each item is rated on a 5-point scale. The score ranges from 0 to 100, with higher scores reflecting greater resilience. Resilience can be viewed as a measure of stress coping ability.[24]

The hospital anxiety and depression scale (HADS) was used to assess anxiety and depression.[25] This scale is divided into 2 subscales, an anxiety subscale (HADS-A) and a depression subscale (HADS-D), both containing 7 items. Each item is rated on a 5-point scale. The Cronbach alpha was .83 for the anxiety subscale and .84 for the depression subscale, indicating adequate internal consistency.[26] The HADS was added to the standard intake procedure in 2009 hence five participants did not fill out the HADS at T0.

The Symptom Check List-90-Revised (SCL-90-R) assesses self-reported psychological distress and multiple aspects of psychopathology. It consists of 90 questions, each item is rated on a 5-point scale. In this study total scale was used as a measure for psychological distress.[27] Internal consistency of the total scale is excellent.[28] The SCL-90-R was added to the standard intake procedure in 2010, hence 9 participants did not fill out the SCL-90-R at T0.

### Statistical procedures

Data was anonymised. Changes in pain scores (intensity of worst and least pain of the past week), domain scores of the WHOQOL-BREF (physical, psychological, and social), resilience scores, and HADS scores (depression and anxiety) were checked for normal distribution. Changes were normally distributed, hence a paired-sample t- test was applied.

We operationalized the outcome variables as follows. A poor outcome regarding pain (the worst pain in the past week) was present if the improvement was <2 points on the NRS.[29] A poor outcome regarding mobility was present if the participant rated the change as less than an “important” improvement. A poor outcome regarding CRPS-I was present if the physician judged CRPS-I to be present (in the residual limb or elsewhere), based on Bruehl criteria. [1]

The following potential risk factors, assessed prior to amputation, were explored, for their association with poor outcomes; low scores on the physical, psychological, or social domains of the WHOQOL-BREF (a score of 1 SD below the mean of all participants), poor resilience, (a score of 1 SD below the mean of all participants), a score >8 on one of the HADS domains, psychological distress (a score of 1 SD above the mean of all participants on the SCL-90-R), childhood adversity, outstanding life events, a psychiatric disorder or history of a disorder, and being involved a lawsuit. Uni variable linear regression analyses were performed for all 11 potential risk factors and 5 baseline characteristics (social status, age, gender, education and pain) as independent variables, with change in worst pain in the past week (before and after amputation) as dependent variable. Dummy variables were made to analyse social status, level of amputation and education. Factors associated (p<0.1) with change in worst pain in the past week, were entered in multi variable regression analysis. The following factors, assessed prior to amputation, were entered: worst pain intensity in the past week, social support and education. All 11 potential risk factors and 5 baseline characteristics, were also analysed non-parametrically for their association with poor outcomes regarding mobility and recurrence. Associations with mobility were analysed using a Mann-Whitney test and associations with recurrence were analysed using Fischer’s exact test. Results are significant at p≤0.05 unless stated otherwise. All analyses were performed in IBM SPSS Statistics (v.22).

## Results

Thirty-one patients, mean (sd) age 41 (12.1), 6 men and 25 women, participated (Table 1).

**Table 1:** Clinical characteristics of 31 participants.

**Variable**	**Mean(SD) T0**	**n(%) T0**
Age (years)	37.5(12.5)	
Women		25(81)
		
Social status		
Living alone		8(26)
Living together		16(52)
Living with parent(s)		7(22)
Education (ISCES level)		
0–4		9(29)
5 and 6		18(58)
7–9		4(13)
Presence of		
Childhood adversity		10(32)
Outstanding life events		16(52)
Lawsuit		2(6)
Psychiatric disorder or history of such a disorder		6(19)
Motivation for amputation request±		
Pain reduction		31(100
Contracture		23(74)
Increase mobility		19(61)
Remove “obstacle”		12(39)
Non-functional limb		8(26)
Wounds		8(26)
Dystonia		3(10)
Duration CRPS-I prior to amputation (years)	7.4(6.9)	
	**Mean(SD) T1**	**n(%) T1**
Age (years)	41.4(12.1)	
		
Level of amputation		
Trans-humeral		1(3)
Trans-radial		1(3)
Trans-femoral		6(19)
Knee disarticulation		10(32)
Trans-tibial		13(42)
Time after amputation(years)	3.9(2.2)	
		

T0 = Prior to amputation, T1 = Follow-up, ISCES = The International Standard Classification of Education

± = More answers possible

At follow up pain scores had reduced, scores on the physical domain of the QOL were improved, and SCL-90-R scores had increased (p<0.05)(Table 2).

**Table 2:** Scores before and after amputation and difference in mean scores in 31 patients.

				95% confidence interval of difference		
Variable	Mean (SD) T0	Mean (SD) T1	Differen- ce (SD)	Lo-wer	Up- per	P*
Intensity of worst pain in past week	8.7(0.9)	5.2(3.0)	-3.5 (3.3)	-2.2	-4.7	< .001
Intensity of least pain in past week	6.1(1.8)	2.5(2.9)	-3.6 (3.3)	-2.4	-4.8	< .001
Quality of life Physical domain	9.4(2.5)	12.7(3.7)	3.3 (3.6)	4.6	2.0	< .001
Quality of life Psychological domain	14.1(2.1)	14.6(3.3)	0.5 (2.5)	1.4	0.5	.329
Quality of life Social domain	13.6(3.8)	14.3(3.0)	0.8 (3.5)	2.1	0.5	.230
Resilience CD-RISC	76.9(9.2)	72.5(17.8)	-4.5 (13.7)	-0.6	-9.5	.081
HADS depression (n = 26)#	5.2 (3.4)	3.4 (4.5)	-1.8 (4.6)	-0.1	-3.6	.063
HADS anxiety (n = 26)#	5.1 (3.1)	4.0 (3.6)	-1.0 (3.3)	-0.3	-2.4	.127
SCL-90-R(n = 22)#	128.7(26.2)	148.7(55.7)	20 (44.9)	39.9	0.1	.049

T0 = Prior to amputation, T1 = Follow-up

* = Significance results of paired-sample t test

# = Number of paired data if less than 31.

Scale range: pain; 0–10, Quality of life domains; 0–20, Resilience 0–100, HADS domains 0–21, SLC-90; 90–360

An overview of potential risk factors and outcomes per patient is presented in Table 3.

**Table 3:** Potential risk factors and outcomes of an amputation in longstanding therapy resistant CRPS-I.

Partici- pant	Risk factors				Outcomes			
	Resi- lience score	Social support	Law- suit	Psychia- tric disorder DSM4	Pain change	Mobi- lity chan-ge	Recur- rence, residual limb	Recur- rence, some-where else
1	76	12	N	N	9	++	N	N
2	66	12	N	N	9	++	N	N
3	67	17	N	N	9	+	N	N
4	77	11	N	N	8	++	N	N
5	82	17	N	N	7	++	N	N
6	69	20	N	N	7	+	N	N
7	71	17	N	Y	7	++	N	N
8	91	17	N	N	7	++	N	N
9	71	16	N	N	6	++	N	N
10	69	15	N	Y	6	+	N	N
11	90	11	N	N	5	++	N	N
12	85	17	N	N	4	++	N	N
13	80	13	Y	N	4	+	Y	N
14	88	16	N	N	4	++	N	N
15	76	15	N	N	3	++	N	N
16	70	9	N	N	3	++	N	N
17	88	20	N	N	3	++	N	N
18	87	8	N	N	2	++	N	N
19	66	12	N	N	2	++	Y	Y
20	69	12	N	HIS	2	+	N	N
21	81	11	Y	N	1	+-	Y	Y
22	76	12	N	Y	1	++	N	N
23	64	12	N	N	1	++	N	N
24	59	9	N	N	1	+	N	N
25	69	11	N	HIS	1	—	N	N
26	83	19	N	N	0	++	Y	N
27	95	19	N	N	0	++	N	N
28	83	11	N	N	0	++	Y	N
29	79	5	N	N	-1	++	N	N
30	71	9	N	N	-2	++	N	N
31	86	15	N	HIS	-2	++	N	N

Resilience score = total score of CD-RISC, Social support = total score of social domain at initial assessment, Y = Potential predictor is present prior to amputation, N = not present HIS = history of psychiatric disorder prior to amputation, Pain change = change in worst pain in past week, higher values indicate larger improvements, Mobility change = mobility change between before and after amputation; ++ = Important improvement, + = small improvement, +- = no change, — = important deterioration, Recurrence according to Bruehl criteria: Y = outcome is present at follow up, N = not present; GRAY SHADED Predictors = potential predictor of poor outcome; GRAY SHADED Outcomes = outcome is poor (see text for operationalisations).

Eleven participants (35%, 95% confidence interval (CI) 21% to 53%) had a poor outcome regarding pain, 8 participants (26%, 95% CI 14% to 43%) had a poor outcome regarding mobility and 12 participants (39%,95%CI 24% to 56%) reported a recurrence. Of these 12 participants 5 (16%, 95% CI 7% to 33%) had a recurrence confirmed by a physician following Bruehl criteria. Seven patients (23%, 95% CI 11% to 40%) had 2 poor outcomes and 1 participant (3%, 95% CI 1% to 16%) had 3 poor outcomes. Reduction of worst pain in the last week was less in participants with a poor social support (Table 4).

**Table 4:** Results of the 2 regression analyses with change in worst pain in the past week as dependent variable. Model 1 without controlling for education, model 2 with controlling for education.

Model		Unstandardized Coefficients		Sig.	95% Confidence Interval for B		Model correlation	
		B	SE B		Lower Bound	Upper Bound	R	R Square change
1	(Constant)	-20.8	5.1	<0.001	-31.1	-10.4	0.679	.461 p<0.001
	Social support	0.4	0.1	0.004	0.1	0.6		
	Pain^a^	2.2	0.5	<0.001	1.1	3.2		
2	(Constant)	-18.8	4.9	0.001	-28.8	-8.7	0.741	.088 p = 0.099
	Social support	0.3	0.1	0.011	0.1	0.6		
	Pain^a^	2.0	0.5	0.001	0.9	3.0		
	Education high^b^	3.3	1.5	0.037	0.2	6.4		
	Education middle ^b^	0.6	1.0	0.563	-1.5	2.6		

a: Worst pain in the past week assessed prior to amputation

b: the reference group for education low education.

Participants with low resilience perceived a less important improvement in mobility score (Mann-Whitney U, Z = -2.398, p = 0.015, median resilience of those with an important improvement n = 23: 79 and median resilience of others n = 8: 69). No other variables were associated with an important improvement in mobility. Twelve participants (38%, 95%CI 24% to 56%) believed they had recurrence of the CRPS-I in the residual limb and 8 (26%, 95%CI 14% to 43%) believed somewhere else. According to Bruehl criteria, 5 participants (16%, 95%CI 7% to 33%) had a recurrence in the residual limb and 2 participants (6%, 95%CI 2% to 21%) also somewhere else.

Being involved in a lawsuit was associated with a recurrence in the residual limb (Bruehl criteria). A psychiatric disorder or history of psychiatric disorder was associated with a recurrence somewhere else (Bruehl criteria) and with reporting a recurrence somewhere else (Table 5).

**Table 5:** Associations of psychological factors and poor outcome of an amputation in longstanding therapy resistant CRPS-I in 31 patients.

	Recurrence	No recurrence	significance
	In residual limb (Bruehl, n = 5)	In residual limb (Bruehl, n = 26)	
Psychiatric^a^ (n = 6)	2	4	0.241
Lawsuit^b^ (n = 2)	2	0	0.022*
	Somewhere else (Bruehl, n = 2)	Somewhere else (Bruehl, n = 29)	
Psychiatric^a^ (n = 6)	2	4	0.032*
Lawsuit^b^ (n = 2)	1	1	0.127
	Patient reported in residual limb (n = 12)	Patient reported in residual limb (n = 19)	
Psychiatric^a^(n = 6)	4	2	0.137
Lawsuit^b^ (n = 2)	2	0	0.142
	Patient reported somewhere else (n = 8)	Patient reported somewhere else (n = 23)	
Psychiatric^a^ (n = 6)	4	2	0.026*
Lawsuit^b^ (n = 2)	2	0	0.060

a) Psychiatric disorder or history of psychiatric disorder prior to amputation

b) Patient was in a lawsuit prior to amputation

* = <0.05 Results of Fischer exact test.

No other associations were found between potential risk factors and outcome variables.

## Discussion

This study focussed on associations between psychological factors before amputation and poor outcomes after amputation because of longstanding therapy resistant CRPS-I. Four risk factors were associated with poor outcomes. Poor social support or lower score on resilience were associated with poor outcomes regarding pain and mobility. Having a psychiatric disorder or a history of a psychiatric disorder or involvement in a lawsuit were associated with recurrence.

Amputation in longstanding therapy resistant CRPS-I is a last option but outcomes can be disappointing. Therefore identifying risk factors associated with poor outcome is highly relevant. The association between lack of social support and pain was more or less expected since lack of social support is also a predictor of worse outcomes in patients with arthritis, chronic pain, and patients with an amputation.[30–33] The fact that social support is beneficial for many patients points in the direction of a more general principal and not specific for CRPS-I. We did not find an association between change in worst pain in the past week and anxiety before the amputation (HADS-A). A prospective study into psychological factors, influencing recovery from CRPS-I found an association between high anxiety scores and poor outcome.[34] The main difference with our study is, that in our study participants suffered from longstanding therapy resistant CRPS-I, while in the mentioned study patients responded to treatment of their CRPS-I. Additionally we used different questionnaires to asses anxiety. Contrary to our assumption no association was found between poor outcomes and childhood adversity or outstanding life events. About one third of the participants, had experienced childhood adversity, and more than half had experienced outstanding life events (including childhood adversity). A high incidence of life events in CRPS-I patients was also found in other studies.[35, 36] Childhood adversity or outstanding life events were found to be factors predisposing for chronic pain.[13, 36, 37] The way people handle stress can be weakened by (prolonged) adversity especially in childhood.[38] However, a subgroup of people benefit from a stressful environment and learn to cope better with stress.[39, 40] In our study most participants had normal to high stress coping ability or resilience. It is possible that participants with childhood adversity and outstanding life events in our study, coped well with adversity. But it is still thinkable that stressful periods might contribute to development of CRPS-I, although we did not find any association between presence of stressful life events and recurrence. Participants with low resilience less often perceived an important improvement in their mobility. Such an association was expected, because resilience is a factor that can influence outcome in physically ill people.[41, 42] One rationale is that disease is a stressful event and the way somebody copes with the stress (resilience or stress coping ability) is influencing the impact of the disease. In a previous cross sectional study in patients with an amputation because of longstanding therapy resistant CRPS-I we found that higher resilience scores were associated with a better QOL and lower psychological distress.[43] In that study we found that the resilience of our participants was above average. We wondered why. It might be that only the most resilient patients with CRPS-I are not giving up on looking for a solution after disappointing treatments and end up in a hospital for an amputation far from their home.

The expected association between depression and poor outcome was not found. This association was reported in studies in patients with CRPS-I and in patients after amputation.[15–17,34] But in a prospective multicenter cohort study an association between depression and development of CRPS-I was not found.[36]

In this study participants having a lawsuit before amputation had a higher chance of recurrence in the residual limb. Previous research reported, that being involved in a lawsuit may negatively impact on chronic pain.[44,45] We did not find a significant association. Although the medical examination confirmed recurrence of CRPS-I in the 2 patients that were in a lawsuit at the time of the intake, 1 participant reported a positive, but not clinical relevant, change in pain of 1 point while the other participant reported, a clinical relevant 4 points improvement after amputation and yet claiming recurrence. It is possible that experienced injustice plays a role in the way they experience their symptoms.

A psychiatric disorder or a history of a psychiatric disorder was associated with reported and observed recurrence somewhere else. Of the 6 participants with a psychiatric disorder or a history of a psychiatric disorder 4 didn’t have recurrence somewhere else. For that reason using a psychiatric disorder or a history of a disorder as a potential risk factor for a poor outcome is not specific enough. Additionally a psychiatric disorder or a history of a psychiatric disorder is not precise since it could be any psychiatric disorder described in the DSM-IV and therefor it has limited value in the decision making process. The reason we analysed this potential risk factor, beside depression, anxiety and psychological distress, was the assumed role of a psychiatric disorder in the development of CRPS-I.[6–10] However several reviews could not confirm such a role.[36, 46] Prior to the amputation 3 participants (Table 3: participant 2, 7 and 16) had a potential risk factor for a poor outcome, but their mobility improved and pain decreased considerably, indicating that the prediction of outcomes, based on our findings, is currently not specific enough. A possible explanation is that also other factors, psychologically, physically and medically, play a role in outcomes after amputation because of longstanding therapy resistant CRPS-I. Other factors that also influence outcomes are the common therapeutic factors e.g. expectations or a placebo effect.[47, 48]

The risk factors identified in this study are also not sensitive. Four participants had no risk factors but had poor outcome in 1 or more outcomes (Table 3: participant 6, 26, 27 and 28). Table 3 illustrates the lack of clear pattern in associations. As already mentioned, possibly other factors or a cluster of factors not assessed in this study can predict outcomes better, such as pain related fear, catastrophic thinking, coping style or perception disturbance.[34] Patients ruminating about the worst case scenarios (catastrophic thinking) may interpret any bodily feeling as harmful. This mechanism may play a role in reporting of recurrence of CRPS-I (12 patients reported recurrence but CRPS-I was only confirmed in 5 cases by the physician). As a result of this study we added a scale for pain related fear and catastrophizing to our clinical practice. The data of this study do not support the assumed role of psychological factors or psychiatric disorders in the etiology, development and maintenance of CPRS-I. They do support the assumed role of psychological factors in rehabilitation.

### Limitations of this study

A limitation of our study is the presence of ceiling effects of pain scores, 75% of the participants scored 9 or 10 on the NRC scale before amputation. Additionally the time between amputation and follow-up differed between participants (mean 3.9 years (SD2.2)). Other weaknesses are the use of 11 potential risk factors and 3 different outcomes in a small data set with some missing data of which only change in pain was normally distributed resulting in several non-parametric analyses. Some significant associations might be related to multiple testing.

## Conclusion

Poor outcomes of amputation in longstanding therapy resistant CPRS-1 are associated with psychological factors.

These factors are not specific for the recovery or rehabilitation of CRPS-I. Outstanding life events are not associated with poor outcomes although half the participants had experienced outstanding life events.
